# Identifying diagnostic markers and constructing a prognostic model for small-cell lung cancer based on blood exosome-related genes and machine-learning methods

**DOI:** 10.3389/fonc.2022.1077118

**Published:** 2022-12-22

**Authors:** Kun Zhang, Chaoguo Zhang, Ke Wang, Xiuli Teng, Mingwei Chen

**Affiliations:** Department of Respiratory and Critical Care Medicine, First Affiliated Hospital of Xi’an Jiaotong University, Xi’an, Shaanxi, China

**Keywords:** small-cell lung cancer, exosome, machine learning, diagnostic markers, prognostic model

## Abstract

**Background:**

Small-cell lung cancer (SCLC) usually presents as an extensive disease with a poor prognosis at the time of diagnosis. Exosomes are rich in biological information and have a powerful impact on tumor progression and metastasis. Therefore, this study aimed to screen for diagnostic markers of blood exosomes in SCLC patients and to build a prognostic model.

**Methods:**

We identified blood exosome differentially expressed (DE) RNAs in the exoRBase cohort and identified feature RNAs by the LASSO, Random Forest, and SVM-REF three algorithms. Then, we identified DE genes (DEGs) between SCLC tissues and normal lung tissues in the GEO cohort and obtained exosome-associated DEGs (EDEGs) by intersection with exosomal DEmRNAs. Finally, we performed univariate Cox, LASSO, and multivariate Cox regression analyses on EDEGs to construct the model. We then compared the patients’ overall survival (OS) between the two risk groups and assessed the independent prognostic value of the model using receiver operating characteristic (ROC) curve analysis.

**Results:**

We identified 952 DEmRNAs, 210 DElncRNAs, and 190 DEcircRNAs in exosomes and identified 13 feature RNAs with good diagnostic value. Then, we obtained 274 EDEGs and constructed a risk model containing 7 genes (TBX21, ZFHX2, HIST2H2BE, LTBP1, SIAE, HIST1H2AL, and TSPAN9). Low-risk patients had a longer OS time than high-risk patients. The risk model can independently predict the prognosis of SCLC patients with the areas under the ROC curve (AUCs) of 0.820 at 1 year, 0.952 at 3 years, and 0.989 at 5 years.

**Conclusions:**

We identified 13 valuable diagnostic markers in the exosomes of SCLC patients and constructed a new promising prognostic model for SCLC.

## 1 Introduction

Small-cell lung cancer (SCLC) constitutes approximately 15% of lung cancers and is characterized by a very high proliferation rate, susceptibility to early metastasis, and poor prognosis (1). Unlike non-small cell lung cancer (NSCLC), the survival rate of which has gradually increased, SCLC remains stable at a low survival rate of 14% to 15%, with a median survival of< 2 years for early stage SCLC patients and 1 year for metastatic patients (2). The prognosis of SCLC is worse because SCLC usually presents as an extensive disease at the time of diagnosis and lacks effective long-term treatment. Therefore, searching for convenient and sensitive diagnostic markers and new prognostic markers for SCLC are essential avenues to improve the outcome of SCLC patients.

Exosomes are broadly defined as secretory vesicles that “may have a physiological function” (3). Some researchers describe exosomes as extracellularly secreted organelles with a diameter of 30 to 200 nm (4). Exosomes have abundant proteins, lipids, and nucleic acids (5). Almost all mammalian cells can secrete exosomes (6), such as adipocytes (7) and immune cells (8, 9). The role of exosomes during tumor development has been extensively studied, and exosomal RNAs, proteins, and metabolites can influence cellular outcomes through signal transduction (10). In some recent studies on NSCLC, exosomes have shown good diagnostic and therapeutic value. For example, tumor-derived exosomal proteins can be used as diagnostic biomarkers for NSCLC (11), and membrane-bound proteins are also promising prognostic biomarkers (12). In addition, exosomes from distinct cells in NSCLC patients exhibit different functions. NSCLC cell-secreted exosomal circUSP7 was found to promote immunosuppression in NSCLC (13), whereas circulating NK cell-derived exosomes showed antitumor activity (14). However, only a few studies have found that exosomes secreted by SCLC contribute to cancer growth, metastasis, and angiogenesis (15), and some circulating exosomal miRNAs also enhance angiogenesis in SCLC tumors (16). The diagnostic and prognostic role of circulating exosome-related genes in SCLC remains poorly explained.

**Figure 1:**
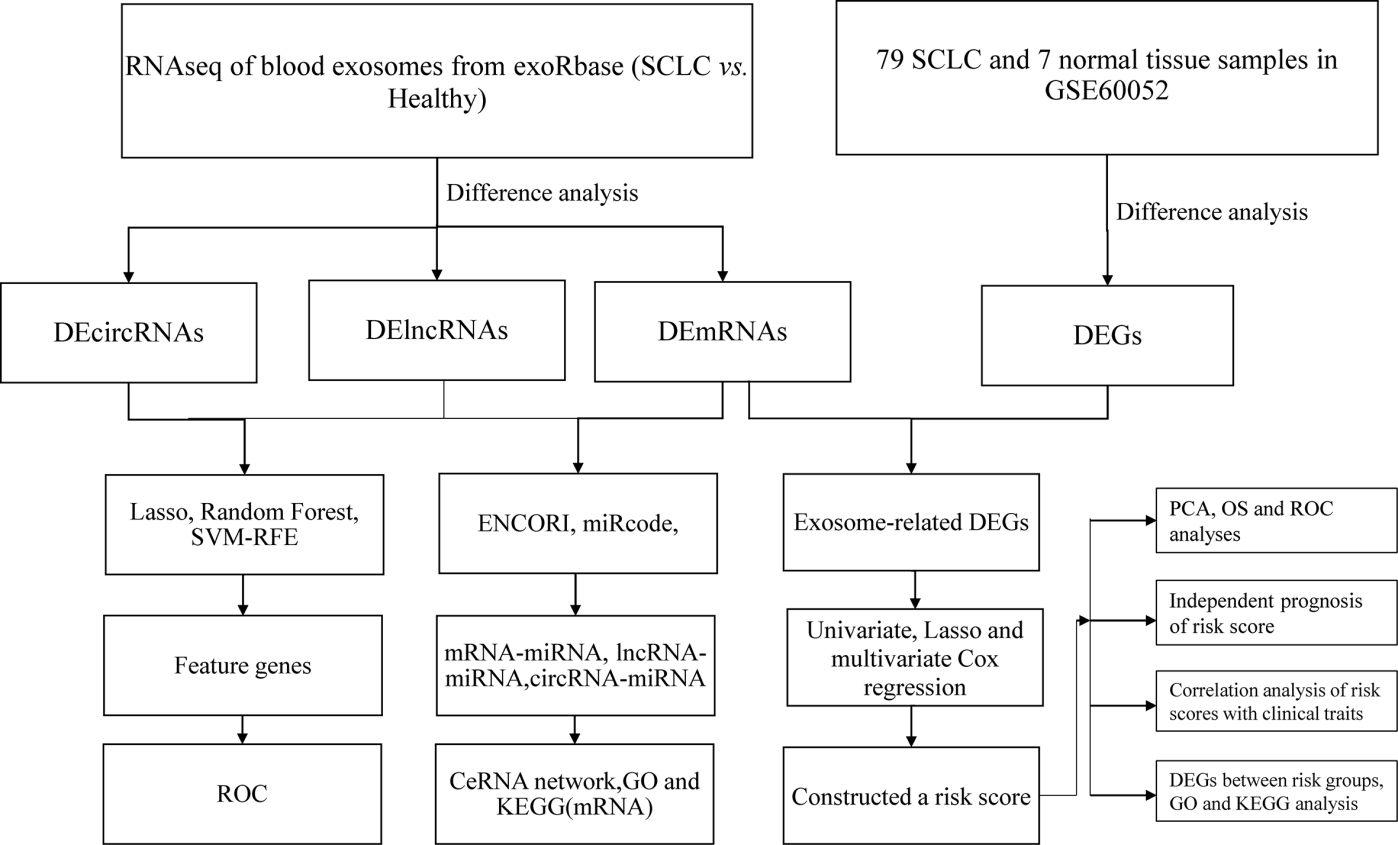
The research flow chart.

Thus, this study aimed to screen valuable diagnostic markers from differentially expressed (DE) RNAs in blood exosomes of SCLC by machine-learning methods and to develop a prognostic model associated with exosome-associated differentially expressed genes (EDEGs). **Figure 1** shows the study’s flow.

## 2 Materials and methods

### 2.1 Data collection

We downloaded RNA sequencing (RNAseq) data (mRNA, lncRNA, and circRNA) of extracellular vesicles (mainly exosomes) in blood from 118 healthy individuals and 36 SCLC patients from the exoRBase 2.0 database (http://www.exorbase.org/) (17). The RNAseq and clinical data of tumor tissues from SCLC patients were extracted from the Gene Expression Omnibus (GEO) database (https://www.ncbi.nlm.nih.gov/theGEO/, GSE60052). The GEO cohort included 7 control and 79 SCLC samples, while only 48 SCLC patients had complete follow-up data (**Table S1**). The baseline information of these two datasets is shown in **Table S2**.

### 2.2 Identification of exosomal DERNAs

In the RNAseq samples from SCLC and healthy individuals, duplicate gene data were averaged using the R package “limma” (18), and the difference analysis was performed using the Wilcox test (19). The DEG filtering criteria were |log2FC| > 1 and false discovery rate (FDR)< 0.05. All analyses were performed with R software (version 4.2.1).

### 2.3 Machine learning to identify exosomal feature DERNAs

The least absolute shrinkage and selection operator (LASSO) (20), Random Forest (21), and support vector machine-recursive feature elimination (SVM-RFE) (22) were used to identify feature DEmRNAs, DElncRNAs, and DEcircRNAs of exosomes in blood, respectively. In LASSO, we calculate lambda.min and obtain the feature genes corresponding to the minimum point of cross-validation error. In Random Forest, the genes with MeanDecreaseGini values greater than 1 are the feature genes. In SVM-RFE, the genes corresponding to the minimum point of cross-validation error are the feature genes. The intersection of these three algorithms’ results is considered the feature DERNAs. We used the R package “pROC” to evaluate the diagnostic values of the feature DERNAs.

### 2.4 Constructing a competitive endogenous RNA (ceRNA) network

The miRNAs interacting with exosomal DERNAs were predicted by the ENCORI (23) and miRcode databases (http://www.mircode.org/). Then, we built the lncRNA-miRNA-mRNA-circRNA network (ceRNA network) (24). The Cytoscape software (v3.7.2) was utilized to visualize the ceRNA network (25). Then, we conducted functional enrichment analysis on DEmRNAs in the ceRNA network.

### 2.5 Construction of the SCLC prognostic model based on EDEGs

DEGs between 79 SCLC samples and 7 normal tissue samples were intersected with exosomal DEmRNAs to obtain the EDEGs. We initially used univariate Cox (uniCox) regression to assess the correlation of each EDEG with the SCLC patient survival. The p-value<0.15 was the prognosis-related EDEG, which was then included in the LASSO Cox regression analysis. Finally, we conducted a multivariate Cox (multiCox) regression of the EDEGs obtained from LASSO Cox and built a risk model ( $\text{Risk Score}=\sum_{i=1}^{N}\text{G}i*Ci\;$ (G: gene expression, C: coefficients)). We divided the SCLC patients into two risk groups based on the median risk score. Survival curves were generated using the Kaplan-Meier method for the low-risk and high-risk groups. The “prcomp” function was applied to conduct the principal component analysis (PCA). In addition, we utilized the “survival”, “timeROC”, and “survminer” R packages to conduct the ROC curve analysis.

### 2.6 Independent prognostic analysis

We combined clinical features and risk score data of SCLC patients and used uniCox and multiCox regression to analyze the independent prognosis of the risk model. In addition, we assessed the differences in risk scores between patients with different clinical characteristics, including age, sex, smoking status, Stage, T stage, and N stage.

### 2.7 Functional enrichment analysis

We performed a differential expression analysis of RNAseq between the two risk groups. The DEG screening criteria were |log2FC| > 1 and p value< 0.05. Then, GO and KEGG analyses and visualization of the DEGs were performed using the R package “ClusterProfiler”.

## 3 Results

### 3.1 Identification of exosomal DERNAs and feature DERNAs

**Figure 2:**
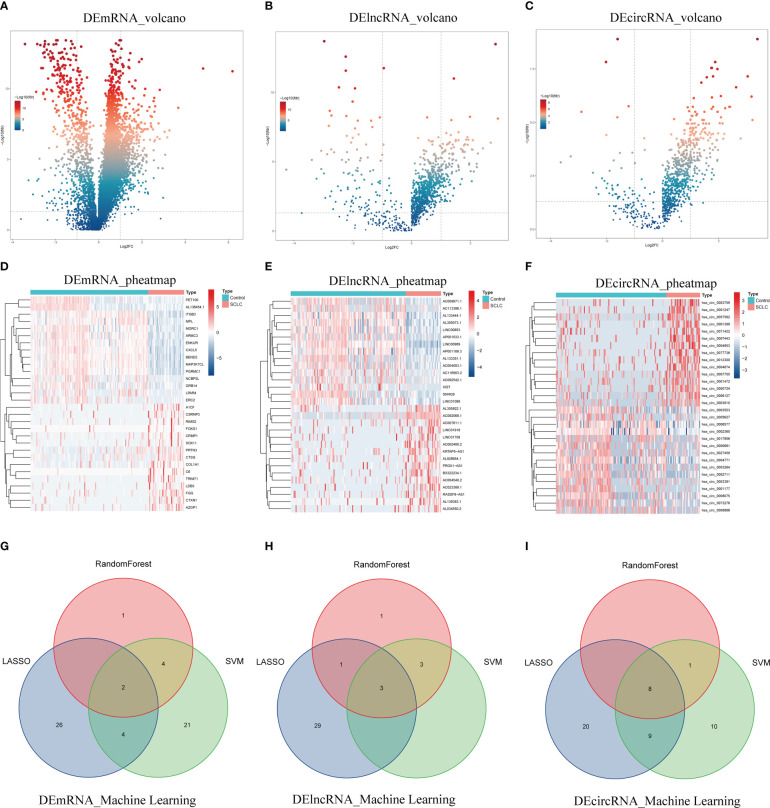
Identification of exosomal DERNAs. **(A–C)** Volcano plots of exosomal DEmRNAs, DElncRNAs, and DEcircRNAs. **(D–F)** Heatmaps of DEmRNAs, DElncRNAs, and DEcircRNAs. **(G–I)** Venn diagrams of machine-learning results for DEmRNAs, DElncRNAs, and DEcircRNAs.

We first identified 952 DEmRNAs (**Figure 2A**), 210 DElncRNAs (**Figure 2B**), and 190 DEcircRNAs (**Figure 2C**) of blood exosome RNAseq between 36 SCLC patients and 118 healthy individuals from the exoRBase database. The top 30 DEmRNAs, DElncRNAs, and DEcircRNAs are shown as heatmaps (**Figures 2D–F**). Three machine learning algorithms, including LASSO, Random Forest, and SVM-REF, were further applied to identify the most valuable 2 DEmRNAs (HIST1H1E, ID2, **Figure 2G**), 3 DElncRNAs (AP000547.3, AC092069.1, AC022150.4, **Figure 2H**) and 8 DEcircRNAs (hsa_circ_0001953, hsa_circ_0002360, hsa_circ_0007443, hsa_circ_0007637, hsa_circ_0005615, hsa_circ_0005455, hsa_circ_0001258, hsa_circ_0000437, **Figure 2I**) from the DERNAs obtained above (**Table 1**, **Figure S1**, **Table S3**).

**Table 1:** Feature DERNAs screened by machine learning.

RNA	LASSO, Random Forest and SVM-RFE
mRNA	HIST1H1E, ID2
lncRNA	AP000547.3, AC092069.1, AC022150.4
circRNA	hsa_circ_0001953, hsa_circ_0002360, hsa_circ_0007443, hsa_circ_0007637, hsa_circ_0005615, hsa_circ_0005455, hsa_circ_0001258, hsa_circ_0000437

DE, differentially expressed.

### 3.2 Diagnostic value of the feature DERNAs

**Figure 3:**
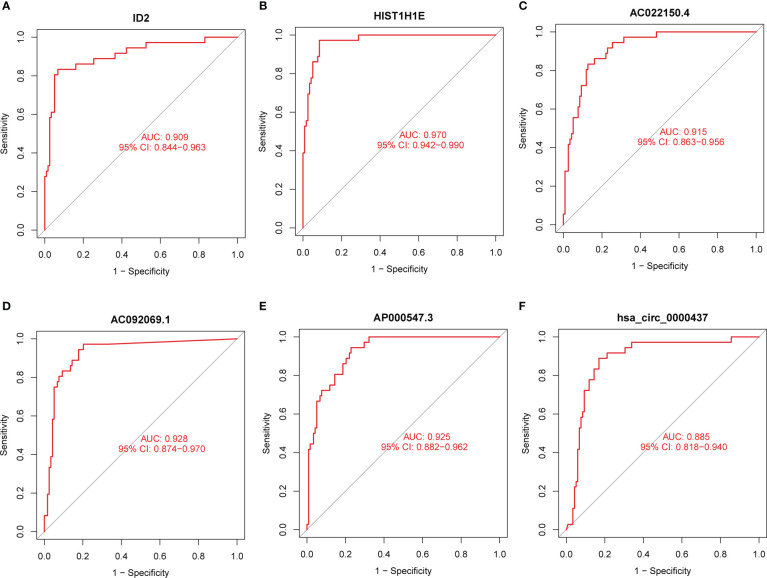
The diagnostic values of the feature DERNAs. **(A)** ROC curve of ID2. **(B)** ROC curve of HIST1H1E. **(C)** ROC curve of AC022150.4. **(D)** ROC curve of AC092069.1. **(E)** ROC curve of AP000547.3 **(F)** ROC curve of hsa_circ_0000437.

To assess the diagnostic value of exosomal feature DEmRNAs, DElncRNAs, and DEcircRNAs, we conducted the ROC curve analysis. The areas under the ROC curve (AUCs) of ID2 and HIST1H1E in DEmRNA were 0.909 (**Figure 3A**) and 0.970 (**Figure 3B**), respectively. The AUCs of AC022150.4, AC092069.1, and AP000547.3 in DElncRNA were 0.915(**Figure 3C**), 0.928 (**Figure 3D**), and 0.925 (**Figure 3E**), respectively. The AUC of hsa_circ_0000437 in DEcircRNA was the maximum (0.885, **Figure 3F**), and the AUCs of the remaining feature DEcircRNAs are shown in **Figure S2**.

### 3.3 Constructing a ceRNA Network

**Figure 4:**
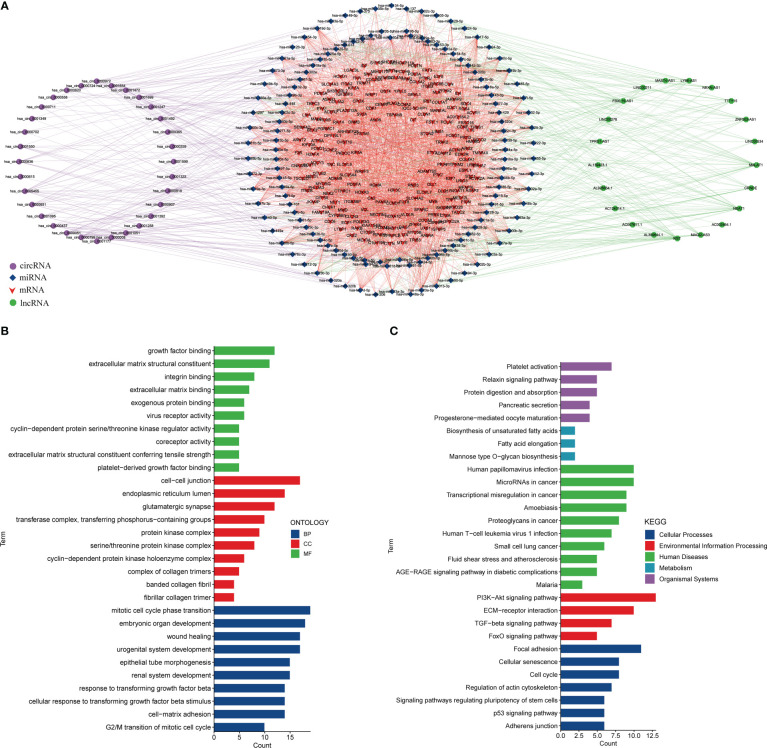
Constructing a ceRNA network. **(A)** The ceRNA network. Left circle: circRNA, middle circle: miRNA and mRNA, right circle: lncRNA. Purple line: circRNA-miRNA, red line: mRNA-miRNA, green line: lncRNA-miRNA. **(B)**. GO analysis of DEmRNAs in the ceRNA network. **(C)** KEGG analysis of DEmRNAs in the ceRNA network.

Meanwhile, we predicted miRNAs that may bind to DEmRNAs, DElncRNAs, and DEcircRNAs and constructed a ceRNA network (**Figure 4A**). The ceRNA network included 198 mRNAs, 21 lncRNAs, 134 miRNAs, and 32 circRNAs (**Table S4**). There were 2 miRNAs interacting with ID2, 19 miRNAs interacting with hsa_circ_0005455, 2 miRNAs interacting with hsa_circ_0001258, and 14 miRNAs interacting with hsa_circ_0000437 (**Table S5**). Then, we performed the functional enrichment analysis of the DEmRNAs in the ceRNA network. The top terms in the biological process (BP) category were growth factor binding and extracellular matrix structural constituent. The top terms in the cell component (CC) category were endoplasmic reticulum lumen and cell-cell junction. In molecular function (MF), these genes were primarily abundant in mitotic cell cycle phase transition and embryonic organ development (**Figure 4B**). The KEGG analysis showed that these DEmRNAs were associated with the PI3K-Akt signaling pathway, ECM-receptor interaction, and TGF-beta signaling pathway (**Figure 4C**).

### 3.4 Construction of SCLC prognostic model based on EDEGs

**Figure 5:**
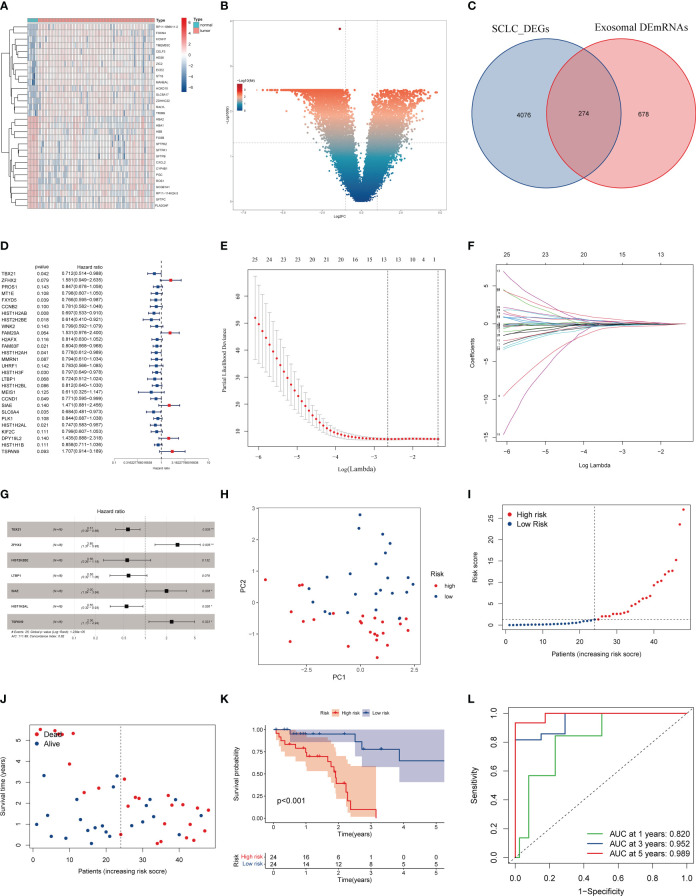
Construction of the SCLC prognostic model based on EDEGs. **(A)** Heatmap of the top 30 DEGs in the GEO cohort. **(B)** Volcano plot of DEGs in the GEO cohort. **(C)** The intersection of exosomal DEmRNAs with DEGs of the GEO cohort. **(D)**. Univariate Cox regression analysis. **(E)** LASSO regression of survival-associated EDEGs **(F)** Cross-validation for LASSO regression. **(G)** Multivariate Cox regression analysis. **(H)** PCA plot. **(I, J)** The median risk score and the distribution of survival time. **(K)** Kaplan-Meier analysis. The numbers in the table represent the numbers of surviving patients. **(L)** ROC curve of the model.

We performed the differential expression analysis on samples from the GSE60052 cohort, yielding 4350 DEGs (**Figures 5A, B**). These DEGs intersected with 952 exosomal DEmRNAs to obtain 274 EDEGs (**Figure 5C**). We then merged these EDEGs expression data with the clinical follow-up data of 48 SCLC patients. **Table 2** presents the clinical data of 48 SCLC patients. Based on the uniCox regression, we obtained 28 prognosis-associated EDEGs (**Figure 5D**). The 28 prognosis-associated EDEGs were then included in the LASSO Cox regression analysis, and 13 representative genes were further screened (TBX21, ZFHX2, MT1E, HIST1H2AB, HIST2H2BE, FAM83F, MMRN1, LTBP1, CCND1, SIAE HIST1H2AL, DPY19L2, and TSPAN9) (**Figures 5E, F**). Finally, we conducted the multiCox regression analysis on the above 13 genes to establish a prognostic model consisting of 7 genes (TBX21, ZFHX2, HIST2H2BE, LTBP1, SIAE, HIST1H2AL, and TSPAN9) (**Figure 5G**, **Table 3**). Here is the formula: risk score = (exp. TBX21* -0.56) + (exp. ZFHX2* 1.05) +(exp. E2F7*0.340957721) + (exp. HIST2H2BE* -0.58) + (exp. LTBP1* -0.54) + (exp. SIAE* 0.69) + (exp. HIST1H2AL* -0.61) + (exp. TSPAN9* 0.86). The median risk score assigned these 48 SCLC patients well into two different risk groups, as confirmed by the PCA analysis (**Figure 5H**). Patients with high-risk scores had higher mortality and shorter survival times than those with low-risk scores (**Figures 5I–K**). Additionally, we utilized the ROC curve analysis to assess the specificity and sensitivity of the risk model, which showed that the AUC was 0.820 at 1 year, 0.952 at 3 years, and 0.989 at 5 years (**Figure 5L**).

**Table 2:** Baseline characteristics of SCLC patients in GSE60052.

	ALL (N=48)	Alive (N=23)	Dead (N=25)
Age, years			
>65	11 (22.9%)	3 (13.0%)	8 (32.0%)
<=65	37 (77.1%)	20 (87.0%)	17 (68.0%)
Sex			
Female	5 (10.4%)	3 (13.0%)	2 (8.00%)
Male	43 (89.6%)	20 (87.0%)	23 (92.0%)
Smoke			
Smoker	33 (68.8%)	17 (73.9%)	16 (64.0%)
No-smoker	15 (31.2%)	6 (26.1%)	9 (36.0%)
Stage			
I-II	16 (33.3%)	4 (17.4%)	12 (48.0%)
III-IV	32 (66.7%)	19 (82.6%)	13 (52.0%)
T			
T1-T2	36 (75.0%)	14 (60.9%)	22 (88.0%)
T3-T4	11 (22.9%)	8 (34.8%)	3 (12.0%)
Unknow	1 (2.08%)	1 (4.35%)	0 (0.00%)
N			
N0	11 (22.9%)	3 (13.0%)	8 (32.0%)
N1-N3	36 (75.0%)	19 (82.6%)	17 (68.0%)
Unknow	1 (2.08%)	1 (4.35%)	0 (0.00%)
M			
M0	46 (95.8%)	22 (95.7%)	24 (96.0%)
M1	1 (2.08%)	0 (0.00%)	1 (4.00%)
Unknow	1 (2.08%)	1 (4.35%)	0 (0.00%)

SCLC, small cell lung cancer.

**Table 3:** Multivariate Cox proportional hazards regression analysis.

Gene	coef	HR	HR.95L	HR.95H	pValue
TBX21	-0.56022	0.571084	0.385641	0.8457	0.005165
ZFHX2	1.048958	2.854674	1.369668	5.949737	0.005118
HIST2H2BE	-0.58483	0.557201	0.260182	1.19329	0.132283
LTBP1	-0.54064	0.582376	0.319466	1.061652	0.077617
SIAE	0.691107	1.995924	1.038662	3.835425	0.038099
HIST1H2AL	-0.60605	0.545503	0.32045	0.928612	0.02556
TSPAN9	0.858402	2.359387	1.127123	4.938864	0.022759

### 3.5 Clinical prognostic analysis

**Figure 6:**
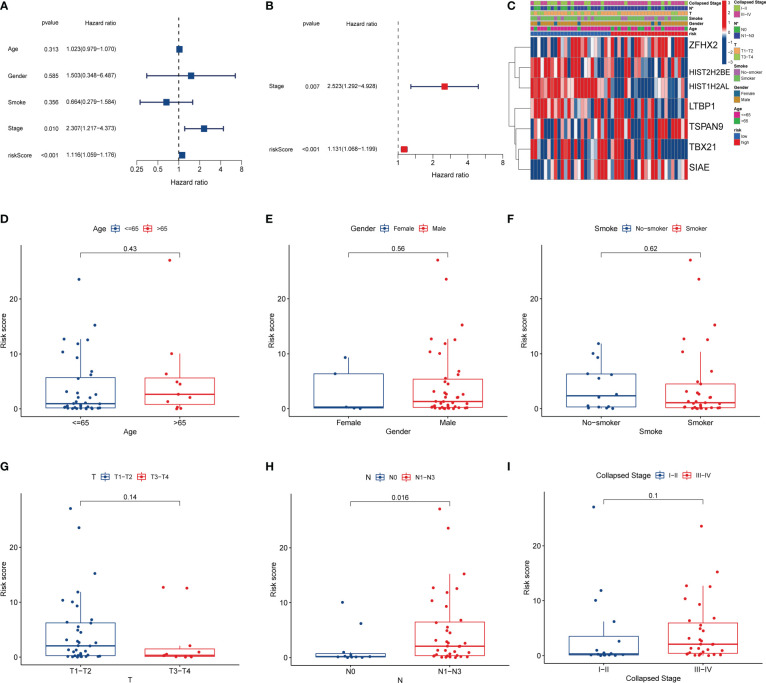
Clinical prognostic analysis. **(A)** Univariate Cox regression analysis. **(B)** Multivariate Cox regression analysis. **(C)** Heatmap of clinicopathological characteristics between the high and low-risk groups. **(D–I)** Correlation of risk scores with clinical features.

We evaluated the independent prognostic value of this risk model using uniCox and multCox regression analyses. The uniCox regression indicated that a higher risk score was related to a lower survival rate (HR=1.116, 95% CI=1.059-1.176, **Figure 6A**). The multiCox regression revealed that this risk model was an independent prognostic indicator for SCLC patients after adjusting for other confounding variables (HR=1.131, 95% CI=1.068-1.199, **Figure 6B**). Moreover, we evaluated the correlation between risk models and clinical features (**Figures 6C–I**). The results showed that N1-N3 stage patients scored higher risk than N0 stage patients, suggesting that this risk model was mainly associated with lymph node metastasis (**Figure 6H**).

### 3.6 Functional enrichment analysis of DEGs between the two risk groups

**Figure 7:**
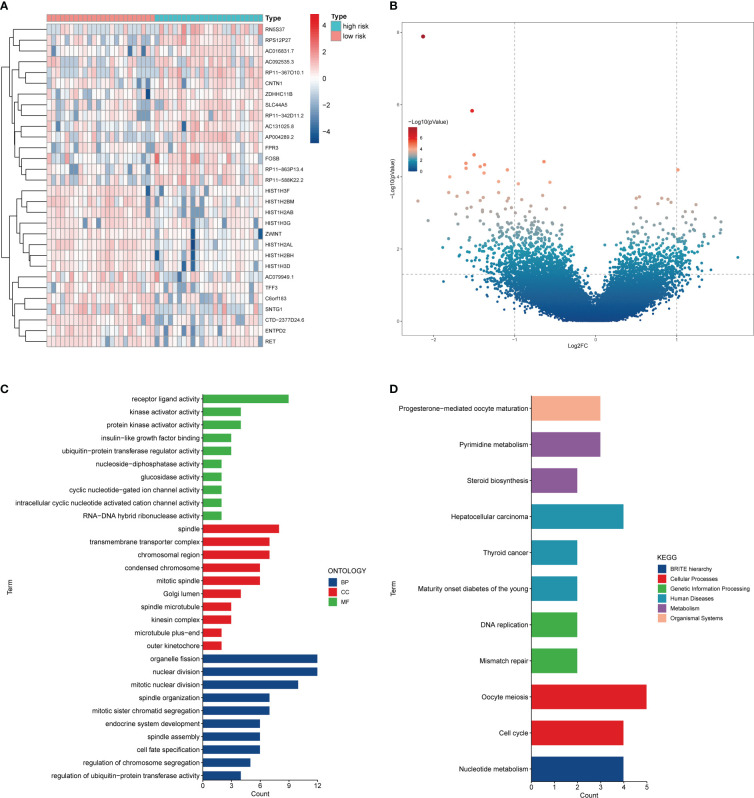
The differential expression analysis between the two risk groups. **(A)** Heatmap of the top 30 DEGs. **(B)** Volcano plot of DEGs. **(C)** GO analysis of DEGs. **(D)** KEGG analysis of DEGs.

Subsequently, we performed the differential expression analysis of RNAseq in SCLC patients between the two risk groups and the functional enrichment analysis of the DEGs. A total of 89 upregulated DEGs and 226 downregulated DEGs were obtained (**Figures 7A, B**). The GO analysis showed that the top BP terms were receptor ligand activity and kinase activator activity, and the top CC terms were spindle and transmembrane transporter complex. In addition, the top MF terms were organelle fission and nuclear division (**Figure 7C**). Meanwhile, the KEGG analysis revealed that these DEGs were associated with the cell cycle, nucleotide metabolism, pyrimidine metabolism, and DNA replication (**Figure 7D**).

## 4 Discussion

As the most aggressive lung cancer type, SCLC has a poor prognosis, and most patients develop metastases at diagnosis (26). Exosomes are a class of secretory vesicles rich in proteins and nucleic acids. Exosomes have a crucial role in immunity, tissue homeostasis, and cancer (4, 27, 28). Several studies have found that exosomes are associated with growth, metastatic angiogenesis, and drug resistance in SCLC (15, 29, 30). However, more research on the role of exosomes in SCLC is needed.

In this study, we initially identified 952 DEmRNAs, 210 DElncRNAs, and 190 DEcircRNAs from blood exosomes and constructed a ceRNA network. Then, we identified the blood exosomal feature DERNAs from SCLC patients by machine learning, and these feature DERNAs showed good diagnostic value. Meanwhile, we extracted EDEGs of SCLC lung tissues by exosomal DEmRNAs. We used EDEGs to construct a prognostic model consisting of 7 genes. The model was mainly associated with lymph node metastasis, and the death risk of SCLC patients increased as the risk score increased. In addition, the model-based prognosis prediction of SCLC exhibited satisfactory efficiency.

Machine-learning algorithms have been increasingly used for automated diagnosis and prognosis prediction in precision medicine (31, 32). Based on 3 machine-learning methods, we identified blood exosomal feature DERNAs of diagnostic value in SCLC patients, including HIST1H1E, ID2, AP000547.3, AC092069.1, AC022150.4, hsa_circ_0001953, hsa_circ_0002360, hsa_circ_ 0007443, hsa_circ_0007637, hsa_circ_0005615, hsa_circ_0005455, hsa_circ_0001258, and hsa_circ_0000437. These RNAs can distinguish SCLC well from healthy individuals with AUC values from 0.781 to 0.970. HIST1H1E is a tumor suppressor whose overexpression inhibits lung cancer cell viability, migration, and invasion (33). ID2 is a transcription factor that is overexpressed in many cancers, such as prostate, breast, and gastric cancers (34, 35). We found that HIST1H1E and ID2 are overexpressed in the SCLC patient blood exosomes. However, the diagnostic value of HIST1H1E and ID2 in SCLC has not been reported. Of the three lncRNAs, only one study reported that AC022150.4 was associated with breast cancer prognosis, but their biological functions in cancer have not been investigated (36). The high abundance, stability, and conservation of circRNAs make them advantageous as diagnostic markers (37). It has been reported that hsa_circ_0002360 is highly expressed in NSCLC tissues and contributes to the malignant behavior of NSCLC (38–40). Circulating hsa_circ_0001953 can be used as a marker for proliferative diabetic retinopathy and active tuberculosis, which have not been investigated in cancer (41, 42). In addition, hsa_circ_0007637, hsa_circ_0005615, hsa_circ_0001258, and hsa_circ_0000437 are associated with the diagnosis and development of nasopharyngeal carcinoma, colorectal cancer, osteosarcoma, and hepatocellular carcinoma (43–46). However, hsa_circ_0005455 and hsa_circ_0007443 have not been reported. Our study provides blood exosomal RNAs with diagnostic value for SCLC patients, which deserves further in-depth study. In addition, we constructed a ceRNA network of exosomal DERNAs, in which ID2 is the target gene of hsa-miR-19a-3p and hsa-miR-19b-3p. Reportedly, hsa-miR-19a-3p can predict the prognosis of lung adenocarcinoma (47), while hsa-miR-19b-3p can be used as a diagnostic and prognostic biomarker for prostate cancer (48). Unfortunately, there are few studies on the role of exosome-derived miRNAs in SCLC.

Our study constructed a model containing 7 prognosis-related genes (TBX21, ZFHX2, HIST2H2BE, LTBP1, SIAE, HIST1H2AL, and TSPAN9) and found it to be a good predictor of OS in SCLC patients. TBX21 is a protein-coding gene specifically expressed in immune cells and highly expressed in tissues such as blood and lung (49). In lung adenocarcinoma, TBX21 expression enhances tumor cell recognition and clearance by the immune system (50, 51), while increased TBX21 expression in cutaneous melanoma is associated with a better prognosis (49). In addition, TBX21 expression in T cells was demonstrated in mouse experiments to control tumor progression and antimetastasis (52). In our study, TBX21 was a protective factor in SCLC, which is supported by previous studies. ZFHX2 encodes a transcription factor with an unknown function that has not been studied in tumors, and we found that its overexpression predicts a poor prognosis for SCLC patients. One study found that high HIST2H2BE levels predicted low survival in NSCLC patients (53). However, we found the opposite result in SCLC patients, with the possible explanation that HIST2H2BE may be tumor-dependent in its molecular status and role. LTBP1 promotes the proliferation of lung adenocarcinoma cells (54), while its high expression in hepatocellular carcinoma inhibits cancer progression (55). The present study identified LTBP1 as a possible suppressor of SCLC. SIAE encodes an enzyme that removes the 9-O-acetylation modification from sialic acid. The SIAE gene regulates Siglec binding in lung cell lines, and NK-mediated cytotoxicity is increased in lung adenocarcinoma cells without SIAE. In other words, high expression of SIAE promotes lung adenocarcinoma progression. Our study found that SIAE is a risk factor for SCLC (56). HIST1H2AL encodes a core histone protein, which was identified in this study with a better prognosis of SCLC (57). However, its specific role in tumors remains unclear and needs further investigation. TSPANs are a family of four transmembrane segments of proteins that may promote tumor growth by affecting angiogenesis and immune function (58, 59). Among them, TSPAN9 has been shown to promote osteosarcoma metastasis (60) and affect the prognosis of breast cancer patients (61). Moreover, we found that high expression of TSPAN9 increases the risk of death in SCLC patients. Overall, the seven genes in our model have yet to be well studied in SCLC and are new prognostic markers for SCLC.

Our study has several advantages. First, multiple machine-learning approaches were used to identify valuable diagnostic markers in the blood exosomes of SCLC patients. Second, we utilized differential genes in blood exosomes to construct a new prognostic model for SCLC with satisfactory predictive effects. This study also has some limitations. The sample size of our SCLC patients was small, and the model’s accuracy needs to be validated in prospective studies with large samples. In addition, *in vitro* and *in vivo* experiments are needed to validate our results.

## 5 Conclusion

In summary, we identified valuable diagnostic markers in the exosomes of SCLC patients by machine-learning methods and constructed a novel promising prognostic model for SCLC using exosome-related genes.

## Data availability statement

The original contributions presented in the study are included in the article/**Supplementary Material**. Further inquiries can be directed to the corresponding author.

## Author contributions

KZ and MC performed study concept and design; KZ, CZ, and MC performed development of methodology and writing; KZ, KW, and XT provided acquisition and analysis of data. All authors read and approved the final paper.

## Supplementary material

Supplementary Figure 1 The results of LASSO, Random Forest, and SVM-REF.

Supplementary Figure 2 The AUCs of the feature DEcircRNAs.
