# Supplementary figures and images for: Identifying diagnostic markers and constructing a prognostic model for small-cell lung cancer based on blood exosome-related genes and machine-learning methods
Source: Front Oncol. 2022 Dec 22;12:1077118. doi: 10.3389/fonc.2022.1077118 (PMC9814973; doi:10.3389/fonc.2022.1077118)

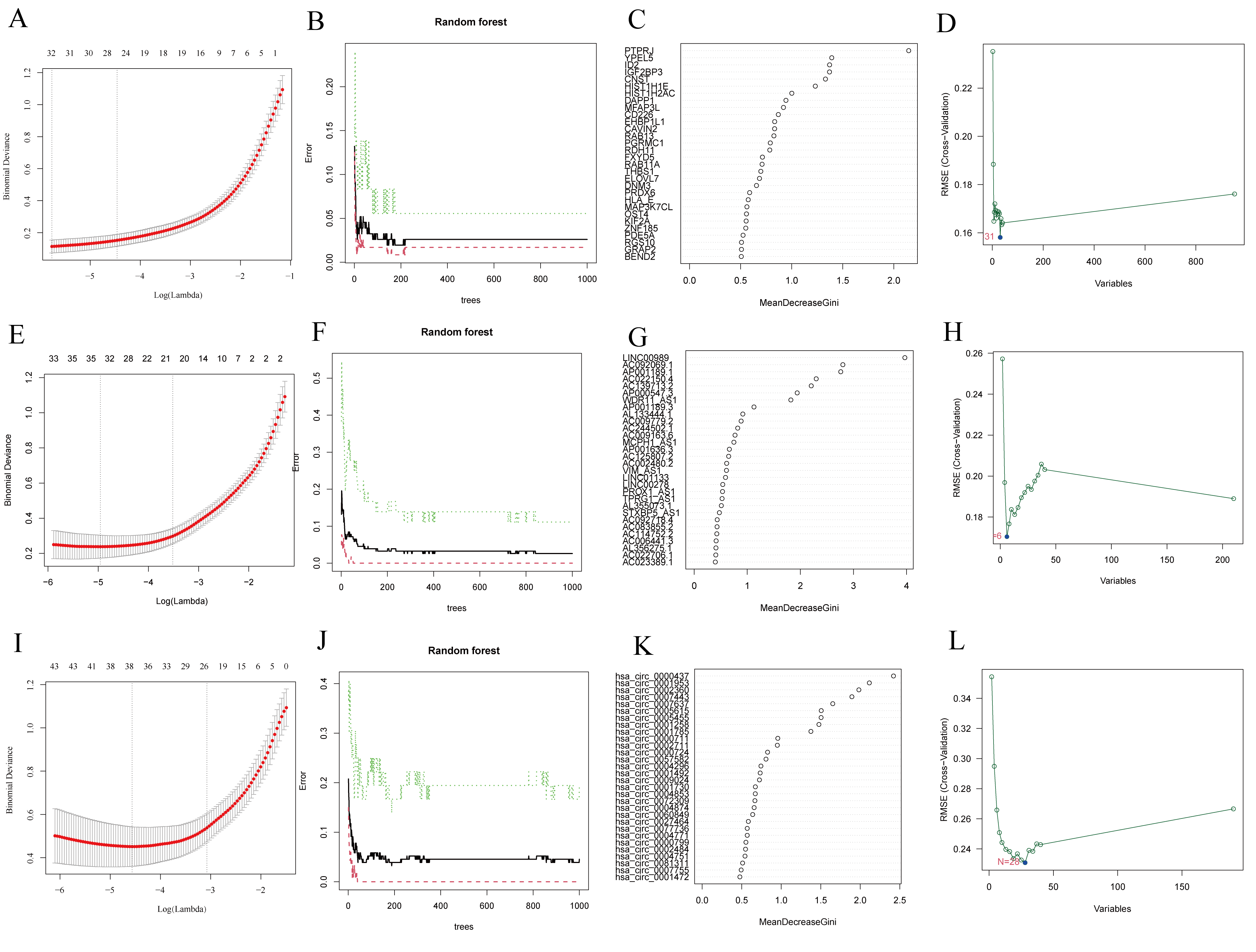

Supplement: Supplementary Figure 1 — The results of LASSO, Random Forest, and SVM-REF. [file Image_1.tif]

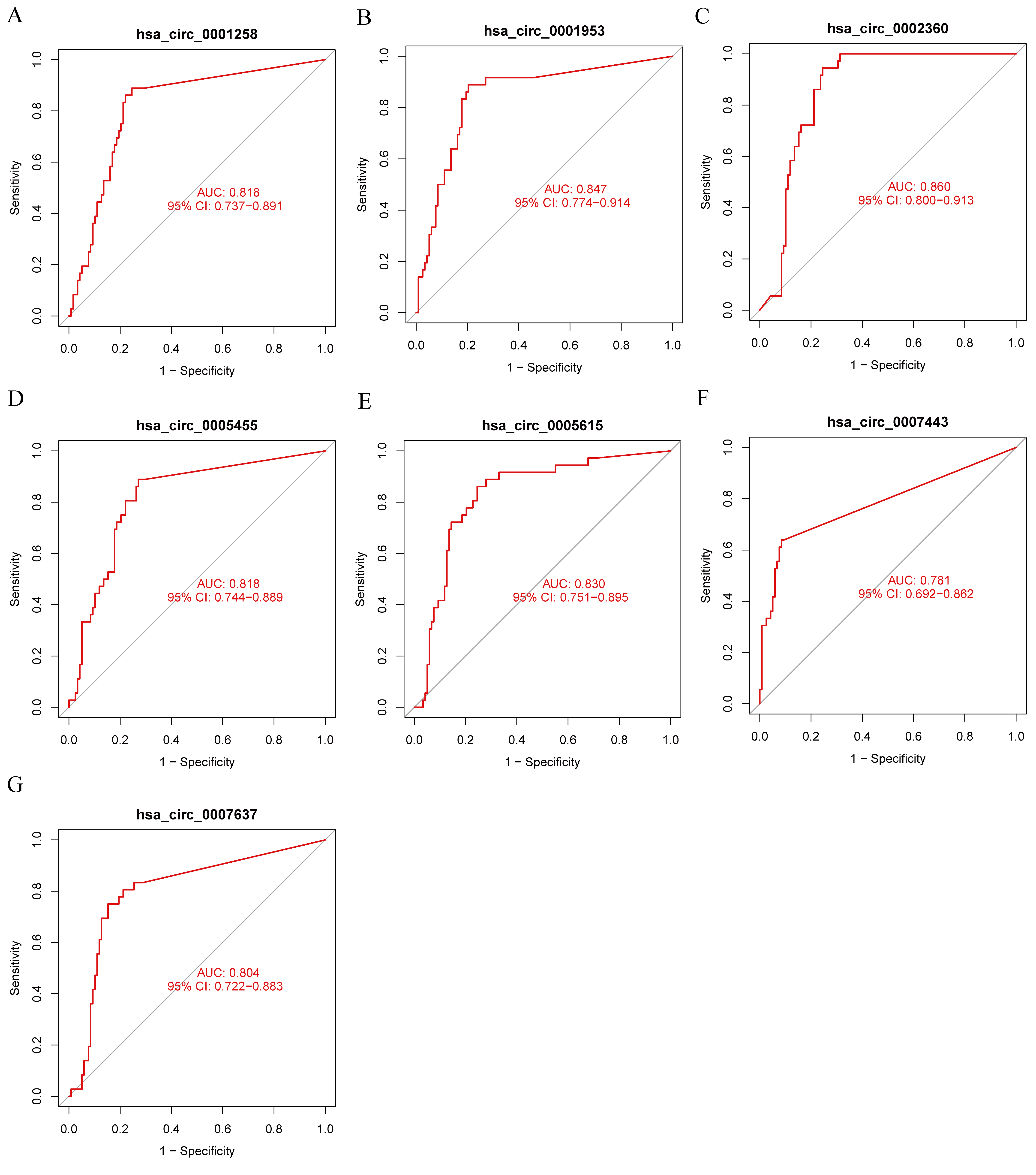

Supplement: Supplementary Figure 2 — The AUCs of the feature DEcircRNAs. [file Image_2.tif]
