# Supplementary material for: Identifying diagnostic markers and constructing a prognostic model for small-cell lung cancer based on blood exosome-related genes and machine-learning methods
Source: Front Oncol. 2022 Dec 22;12:1077118. doi: 10.3389/fonc.2022.1077118 (PMC9814973; doi:10.3389/fonc.2022.1077118)
Supplement: Supplementary file 4 [file Table_2.docx]

| Dataset | Database | Data Type | Sample |
| --- | --- | --- | --- |
| SCLC blood exosomes | exoRBase | RNA-seq | blood exosomes of 118 healthy individuals and 36 SCLC patients |
| GSE60052 | GEO | RNA-seq | Lung tissue of 7 control and 79 SCLC samples |

**Table S2. The baseline information for datasets.**
